# Second harmonic generation from bound-state in the continuum-hosted few-layers van der Waals metasurface

**DOI:** 10.1515/nanoph-2024-0630

**Published:** 2025-01-16

**Authors:** Naseer Muhammad, Azra Begum, Zhaoxian Su, Lingling Huang

**Affiliations:** School of Optics and Photonics, Beijing Engineering Research Center of Mixed Reality and Advanced Display, 47833 Beijing Institute of Technology, Beijing 100081, China

**Keywords:** transition metals dichalcogenides (TMDs), metasurface, second harmonic generation, high-Q, bound-states in the continuum

## Abstract

Monolayer transition metals dichalcogenides (TMDs) have been coupled to bound-state in the continuum (BIC) hosted dielectric structures to attain high second harmonic generation (SHG). However, the transvers electric modes are strongly localized in the waveguides result in fairly weak exciton-photon coupling in monolayer TMD placed on the surface. To achieve SHG in few-layers TMDs based BIC-inspired structure is a challenge. Here, we report BIC in few-layers TMDs metasurface with high quality factor (Q-factor), tunability, and modes-upholding in different environments. The metasurface sustains BIC at different thickness of the meta-atoms, which is highly desired for maintaining the accuracy in fabrications. Next, we calculate the SHG efficiency from few-layers TMD metasurface around BIC wavelengths. The high conversion efficiency in this work is 1.47 × 10^−4^ for 6 mW incident power. Moreover, our design is highly thin and can be used for various linear and non-linear applications in optics. This study will provide a new route to next generation post-silicon metasurfaces.

## 1 Introduction

Metasurfaces are the two dimensional arrangement of artificially engineered meta-units, attracted enormous attention of researchers due to its incredible control of light [1], [2], [3], [4], [5]. The industrial applications of metasurfaces facing challenges due to incompatibility of materials, such as noble metals are incompatible with fabrication of complementary metal-oxide semiconductor. Also, metallic metasurfaces possesses low quality factor (Q-factor) and damage threshold which restrict both linear and nonlinear applications. All-dielectric metasurfaces, silicon in particular bridges this space, although amorphous silicon facing process challenges, robustness and environmental variations [1], [3], [4]. In addition, due to crystal centro-symmetry of the silicon it is not suitable for second harmonic generation (SHG) which is arguably the most prominent nonlinear effect [6].

Bound-states in the continuum (BIC) emerged as a building block of high-Q optics with infinite Q-factor in ideal cases and giant Q-factor in practical structures or quasi-BIC (q-BIC) [7], [8], [9], [10], [11], [12], [13]. Such high-Q metasurfaces have been reported with tremendous improvements in applications including lasing [14], [15], sensing [16], chiral emission [8], [17], [18], [19], [20], image tuning [21], second, third and high harmonic generations [21], [22], [23], [24], [25], [26] to name a few. SHG has been demonstrated in all-dielectric and hybrid configurations of monolayer transition metals dichalcogenides (TMDs) with other materials [27], [28]. However, in such combinations the transverse electric modes can be strongly localized in waveguide, which weakly couple to the monolayer TMD. The Bloch surface waves can effectively excite the monolayer-dielectric based metasurface, because in this case the electric field is confined at the surface of dielectric that strongly couples to the thin films [29]. In these approaches, the transfer of monolayer TMDs on dielectric can introduce in-homogeneities. In addition, strain in TMD layers and topographic irregularities can cause the suppression of nonlinear effects and spectral shifts of excitons [30], [31].

Symmetry protected and accidental BICs have been reported in isolated structures, metasurfaces, different materials and combinations of materials including Si [21], [27], GaAs [11], and multilayer TMDs [10], [32]. Tuning of Q-factor in BIC has been achieved by altering the unit cells and Brillouin zone folding in different metasurfaces. In multilayer TMDs the optical modes and exciton effect team-up to originate BIC in metasurface [10], [32]. The BIC sustain the modes and high Q-factor after transforming it to q-BIC by breaking the symmetry of the resonator [32]. Recently, a strong Rabi splitting was reported in multilayer TMD enabled by so-called BIC in a linear metasurface [10]. SHG from WS_2_ and single MoS_2_ bulk disks have been reported with high intensity [22], [33]. Although, BIC-hosted few-layers TMD structures for nonlinear applications remained unexplored.

In this article, we computationally investigate BIC in ultra-thin few-layers TMDs hetero-symmetric resonator. The few-layers TMD sustain BIC in suspended cases with homogeneous background of *n* = 1.0 and non-homogeneous background. In non-homogeneous, the background is filled with (Silica *n* = 1.45 & PMMA *n* ≈ 1.49). The structure also maintains BIC when placed on a bi-layer substrate. To originate BIC in TMD resonators the optical modes team-up with excitons unlike geometrical optical modes in dielectric. The TMD based resonators are highly tunable, this can be observed from BIC at different thickness or number of layers. We also calculated the SHG conversion efficiency around the BIC wavelengths which is 1.47 × 10^−4^ the highest in this work. The few-layers structure upholds high Q-factor, tunability and modes in all cases and polarizations. Our ultra-thin design can also be used for linear nanophotonic applications and can open a route to new-generation ultra-thin metasurfaces.

## 2 Design, physics and simulations

**Figure 1:**
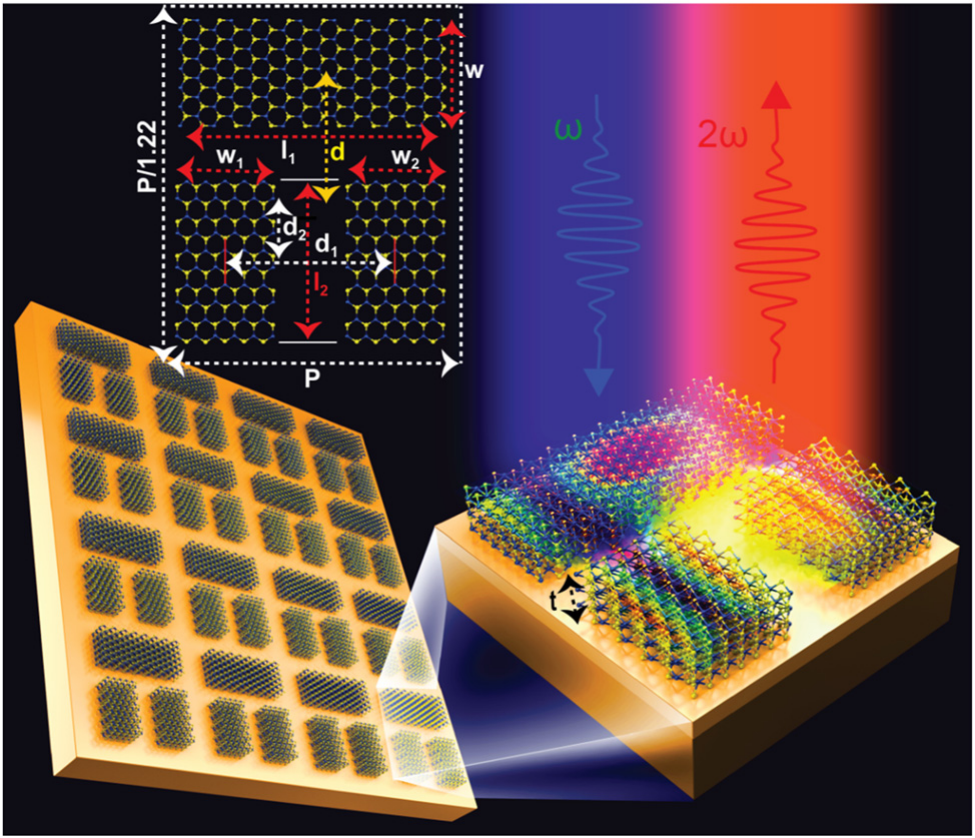
Schematic diagram of pi-shaped TMD metasurface. *p* = 720 nm, *l*_1_ = *p*/1.4, *w* = *p*/5.2, *l*_2_ = *p*/3, *w*_1_ = *w*_2_ = *p*/5, *d* varies from *p*/4.5 to 6, *t* varies from *p*/32.5 to 35, *d*_2_ = 5.3, *d*_1_ = 2*(*p*/4), *t*_1_ = 50 nm, and *t*_2_ = 500 nm.

The rectangular unit-cell comprises of three bars made of WS_2_ arranged in a hetero-symmetric “pi” shaped structure as shown in Figure 1. The two short bars are of same size and the third bar is thin in width and longer in length compared to the two bars. The real part of WS_2_ in-plane permittivity in visible range exceeds 16 and declines gradually in the near-infrared. The refractive index is *n* = ⊡*ɛ*_1_ > 4, significantly dependent on the layer numbers, which is larger than conventional dielectric high index materials including silicon and GaAs [10], [34], [35]. The real part of the dielectric constant *ɛ*_1_ is correlated to imaginary part *ɛ*_2_ which fundamentally related with inter-band transition [34], [36]. The layer dependence of the dielectric constant of TMDs can be examined by following equation as [37]:

$$\varepsilon_{2}\left(\omega \right)=\frac{4\pi^{2}e^{2}}{m_{0}^{2}\omega^{2}}J_{cv}{\left|p_{cv}\right|}^{2}{\left|U\left(0\right)\right|}^{2}\frac{\Gamma /2}{{\left(E_{cv}-h\omega \right)}^{2}+{\left(\Gamma /2\right)}^{2}}$$

where *ω* is frequency, *m*_0_ and *e* are the charge and mass of free electrons, *h* is the Planks constant, *J*_cv_ is the joint density of valence and conduction state, the optical matrix element *p*_cv_ represents the probability of the transitions between the two states, Γ is the damping of transition, *E*_cv_ is optical energy gap, *U* is the influence of exciton on the oscillator strength, 0 is the physical overlap of the hole-electron wave-function, and |*U*(0)|^2^ indicates the effects of excitons on the oscillator strength of inter-band transition. The $\left(E_{cv}-\mathrm{h\omega}\right)=0$ when considering the dielectric constant at the resonance positions thus, the above equation can be written as: $\varepsilon_{2}\left(\omega \right)=\frac{4\pi^{2}e^{2}}{m_{0}^{2}\omega^{2}}J_{cv}{\left|p_{cv}\right|}^{2}{\left|U\left(0\right)\right|}^{2}\frac{2}{\Gamma }$ the Γ and *p*_cv_ are independent on layer-number and respectively indicates the resonance damping and optical matrix element. The later one is related to the unit-cell and its wave-functions. The two parameters, joint density of states and excitonic effect may result in the dependence of dielectric constants on number of layers. All the terms which are independent on the number of layers can be replaced with *A*_0_ in equation. So, the equation can be further simplified as [37]: $\varepsilon_{2}\left(\omega \right)=A_{0}J_{cv}{\left|U\left(0\right)\right|}^{2}$ . It was recently reported that the WS_2_ support whispering galleries, cavity modes and gain of approximately 0.89, which is essential for lasing threshold reduction [35].

The multi-layer structure is highly suitable for gain volume because high carrier density is required for direct-to-indirect band-gap transition due to bandgap re-normalization which is not suitable for lasing. In TMDs the quantum confinement is along the two dimensional plane which leads to different physical properties from their multilayer or bulk forms. This in-turn results in thicknesses of the structure smaller than their resonant wavelengths. Thus, electromagnetic field can be strongly confined in ultra-small volume than conventional dielectric and metallic structures [10], [32], [33], [34], [35], [38], [39]. These fields trapping and modes accumulations have been discussed in comprehensive studies carried out using WS_2_ structures [34], [40], [41]. They demonstrated that TMDs can be fabricated as a dielectric antenna and modes accumulated in TMDs are originated by teaming-up optical modes with excitons [32], [34], [40], [41]. In TMDs metasurfaces BIC can also be achieved using the self-hybridization in bulk structures [10], [40].

TMDs as two dimensional materials demonstrate a range of nonlinear optical responses including SHG, third harmonic generation (THG), two-photon absorption, saturable absorption, and four wave mixing due to their tunable band structures and combinations of chemical compounds [33], [39]. Third order nonlinearity is not restricted due to materials structure, however, SHG is absent in silicon and TMDs with inversion symmetry. The inversion symmetry in TMDs can be broken by using odd-numbered few-layers stacked structures or strain for high second order susceptibility which can be higher than common dielectric such as GaAs [33], [39], [42], [43]. In TMDs, the THG effects are higher compared to SHG under the same applied power. Although, SHG spectras can also be engineered by exciton-Mie coupling or introducing a range of strain values [22], [30], [33], [38].

When the structure is consist of two meta-atoms placed at a certain distance and tuned to maintain a phase shift of an integer multiple of 2*π*, Temporal coupled-mode theory can be used to model a BIC [7], [44], [45] in a system of two modes with amplitudes $A={\left[a_{1}\left(t\right),a_{2}\left(t\right)\right]}^{T}$ evolve in time $\frac{\partial A}{\partial t}=HA$ with Hamiltonian $H=\left[\begin{array}{cc} \omega_{1} & \kappa \\ \kappa & \omega_{2} \end{array}\right]-i\left[\begin{array}{cc} \gamma_{1} & \sqrt{\gamma_{1}\gamma_{2}}{\text{e}}^{\text{i}\psi }\\ \sqrt{\gamma_{1}\gamma_{2}}{\text{e}}^{\text{i}\psi } & \gamma_{2} \end{array}\right]$ where *ω*_1_ *& ω*_2_ indicates the resonant modes and *γ*_1_ *& γ*_2_ represents the damping of modes, *κ* is coupling-factor and the phase-shift is denoted by *ψ*. The via-the-continuum $\left(\sqrt{\gamma_{1}\gamma_{2}}\right)$ is the interference of the two modes radiating into the same channel. One of the eigenvalue evolves into real with infinite life-time and the second one turns to more radiative when it fulfills the condition of $\left(\omega_{1}-\omega_{2}\right)\kappa ={\text{e}}^{\text{i}\psi }\sqrt{\gamma_{1}\gamma_{2}}\left(\gamma_{1}-\gamma_{2}\right)$ . The BIC is achieved when the modes *ω*_1_ = *ω*_2_, *γ*_1_ = *γ*_2_ are alike [7], [45].

## 3 Results and discussions

**Figure 2:**
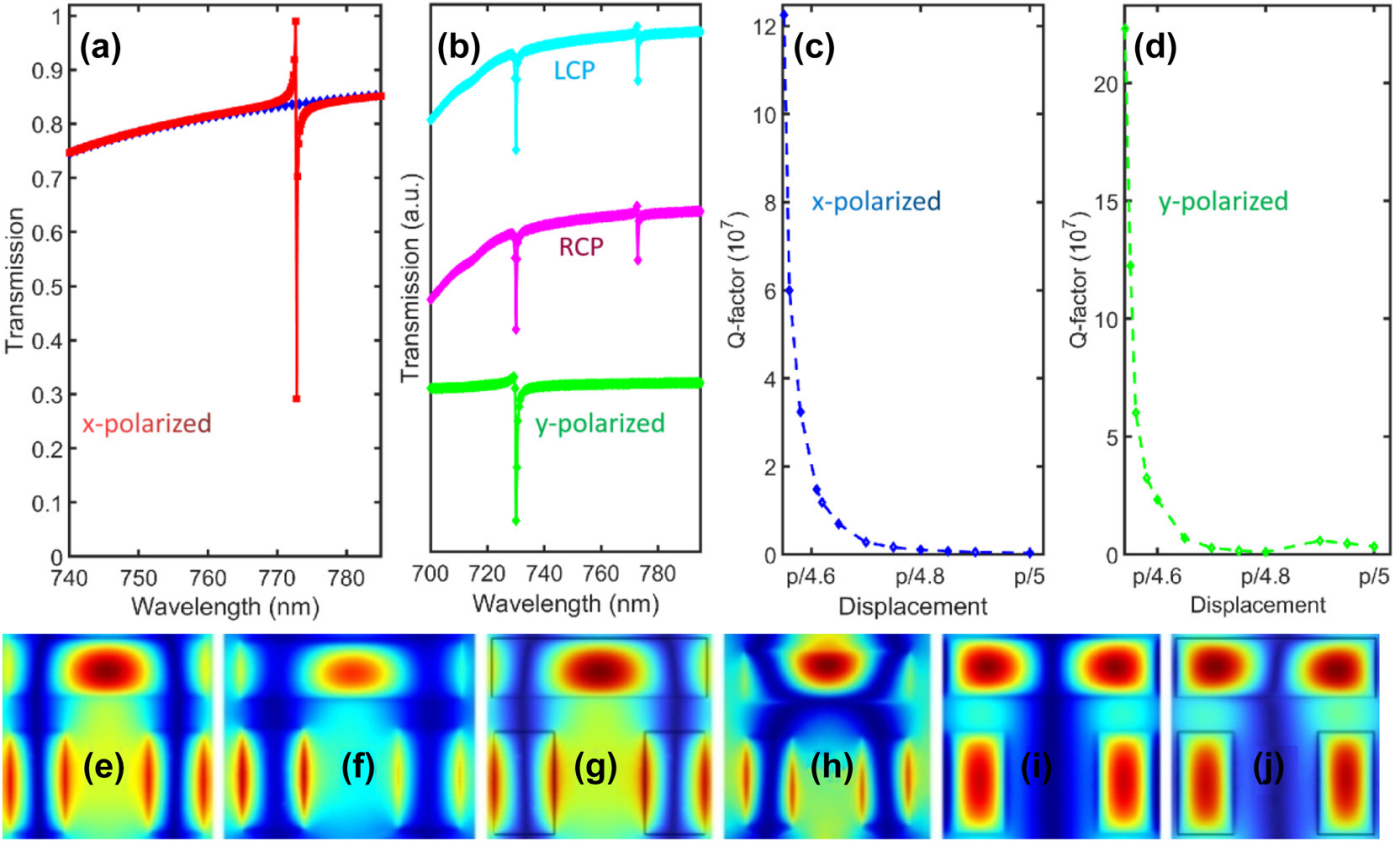
Transmission spectra of suspended structure at (a) displacement *d* = *p*/4.5 and *p*/5.2 under *x*-polarized electric field, (b) displacement *d* = *p*/5.2 *y*-polarized, RCP and LCP, (c) radiative Q-factor for *x*-polarized, and (d) radiative Q-factor for *y*-polarized (for RCP and LCP the radiative Q-factor is the same as *y*-polarized case), (e–h) magnetic field distribution for *x*- and *y*-polarized, RCP and LCP for the q-BIC mode around 772 nm, and (i, j) *y*-polarized and RCP for mode around 730 nm.

First of all, we consider the suspended few-layers TMD (∼22 nm ≈ 33 layers) structure comprised of two parallel short bars and one long bar at a displacement of *d* = 160 nm from the center of the unit-cell as shown in Figure 1 schematic. We apply *x*-polarized incident electromagnetic field and calculate the spectra as shown in Figure 2a (blue curve). The structure completely confine field around 772 nm and does not manifest itself in the transmission spectra because the modes accumulated by bars completely cancel each other. When the long bar is at 160 nm distance from the center of the unit-cell, the two short bars act as perfect mirrors at 300 nm spacing and trap the light in the gap between them. At this distance the structure is tuned to achieve a certain phase shift. The two short bars accumulate two identical modes and completely cancel each other. The influence of the long bar is low and due to weak coupling, the mode trapped by the two short bars cannot be perturb to transform it to leaky mode at *d* = 160 nm.

Next, we move the long bar closer to the two short ones which increases the coupling between the bars and result in a q-BIC mode around 772 nm as shown in Figure 2a (red curve). At displacement *d* = 160 nm the mode possesses the Q-factor up to infinity and decreases as we move the long bar closer to the short ones which can be seen in Figure 2c. Further, we calculate the transmission spectra at different electric field polarization as shown in Figure 2b. In case of *y*-polarized light the Fano-bud of the mode around 772 nm at *d* = 155 nm has a small modulation depth as compared to *x*-polarized. The mode sustains the Q-factor up to infinity and deceases with reducing the space between the long and short bars as shown in Figure 2d. The Q-factor demonstrates similar trend as in *x*-polarized case.

In green spectra, a split appears around 730 nm with large modulation depth. However, this is a low Q-factor leaky mode compared to q-BIC around 772 nm. In case of the left-circularly-polarized (LCP) and right-circularly-polarized (RCP) incident fields, both q-BIC demonstrate large modulation depths with high Q-factor values. In both LCP and RCP a leaky mode appears around 730 nm same as in *y*-polarized. To see the physical mechanism of the BIC mode we calculate magnetic field distribution at the BIC-wavelengths for all four cases. It can be seen from Figure 2e–h that few-layers TMD sustain a clear shape for all four cases with no significant changes. The magnetic field profile at leaky mode around 730 nm display the same distribution for both *y*- and circularly polarized incident beams as shown in Figure 2i and j.

**Figure 3:**
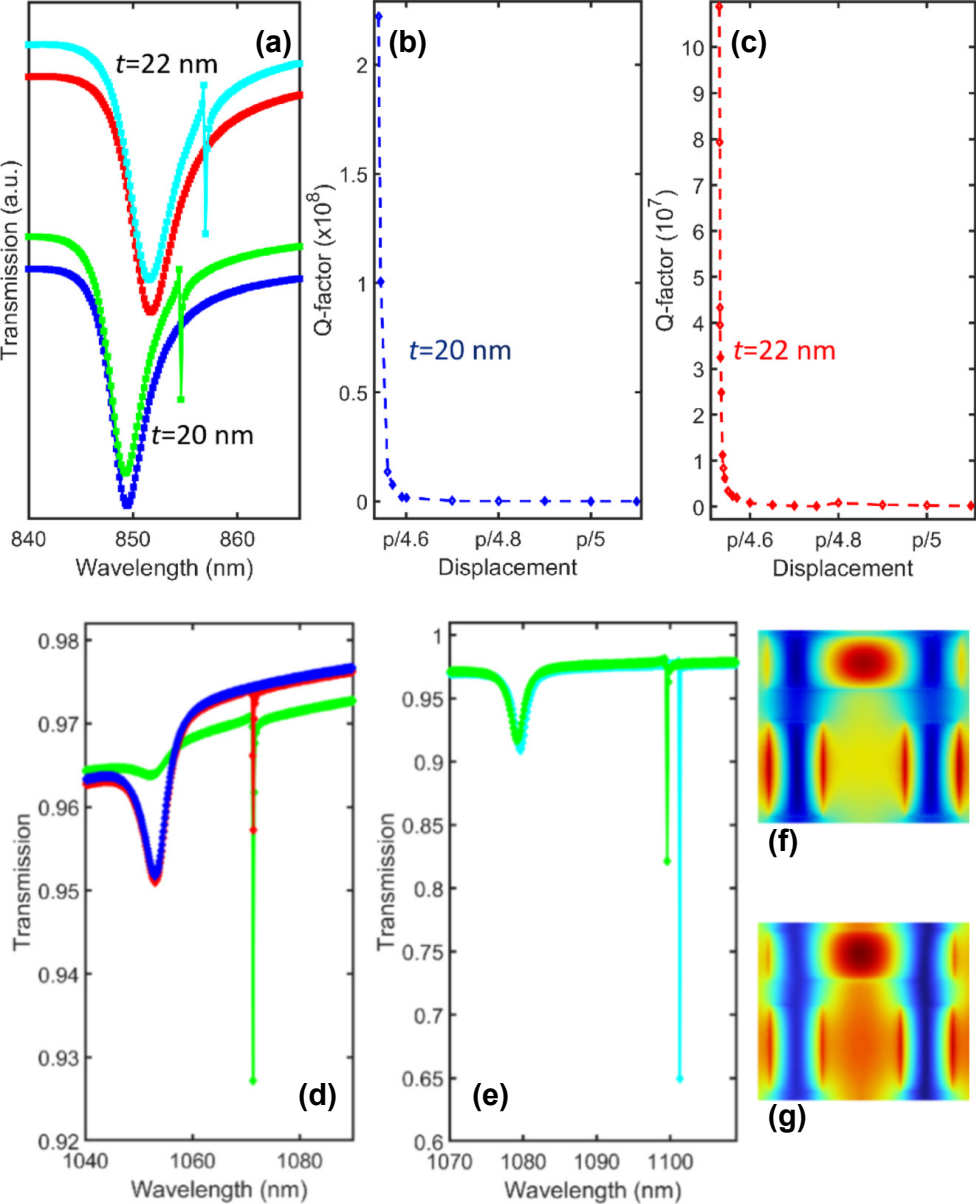
Optical features of bi-layer and infinite substrate cases. (a) Transmission spectra of TMD resonator for *t* = 20 nm and *t* = 22 nm. The structure is placed on the top of 500 nm substrate having refractive index of *n* = 1.5, and 50 nm spacer of *n* = 1.45, between substrate and resonator (metasurface on bi-layer substrate). The radiative Q-factor for (b) *t* = 20 nm and (c) *t* = 22 nm. (d) Transmission spectra at displacement *d* = *p*/4.5 (blue curve, for *x*-polarized the transmission spectra is same) and *p*/6 under LCP (red curve) and *x*-polarized (green curve) case. (e) Transmission spectra at displacement *d* = *p*/6.5 and *w*_*1*_ = *p*/5 (sky blue), and at displacement *d* = *p*/6.5 and *w*_*1*_ = *p*/6 (green). (f) Magnetic field distribution calculated for qBIC in (a), and (g) Magnetic field distribution calculated for sky-blue curve in (e). The structure is placed on infinite SiO_2_ and covered with infinite PMMA 950 (both materials are from Comsol library) in (d) and (e).

To investigate the influence of resonator thickness and substrate on BIC modes we place few-layers TMD metasurface with two different resonator thicknesses (∼31 layers and ∼33 layers) on the bi-layer substrate (a spacer and substrate) as shown in schematic. The modes shift to longer wavelengths due to high refractive index of substrate compared to air in suspended cases. In both cases the metasurface sustain the BIC modes as shown in Figure 3a without a Fano-bud in the transmission spectra. As we decrease the space between the long and short bars BICs transform to q-BICs. In bi-layer substrate case the q-BIC is achieved when the longer bar is at *d* = 141 nm from the center. The radiative Q-factor in both cases is inclined to infinity and decreases with space mediation between the long and short bars, as shown in Figure 3b and c. It can be seen from magnetic field distributions (Figure 3f) the structure maintains the mode shapes similar as in suspended cases (Figure 2e–h). This is in sharp contrast with common dielectric materials where the Q-factor decreases and modes deteriorate with incorporating the substrate in BIC-hosted thin meta-atoms [21]. It is worth noting that in both thickness cases the few-layers metasurfaces demonstrate BIC with same inclined Q-factor. This can be of great importance in fabrication to avoid the modes deterioration with variations in thickness and roughness of the metasurface.

Furthermore, for practical structure we replace the bi-layer substrate with infinite silica and the background air with infinite PMMA on the top of the metasurface which embed the resonators. As the surrounding refractive index increases, the BIC and q-BIC shifts to longer wavelengths around 1,072 nm as shown in Figure 3d. To observe q-BIC we bring the longer bar closer to the short bars at *d* = 120 nm which also contribute to the shift. It is due to the fact that by reducing the gap between the two-short and long bar, the modes accumulated by bars interfere strongly and making circumference like a single aperture. This can be observed from the magnetic field distribution (Figure 3g) where the localized field intensity between the bars is much higher compared to suspended and bi-layer substrate cases. In addition, the resonance intensity of q-BIC increases and spectral width decreases. This can be due to the reason that the coupling between all multipoles is stronger compared to suspended and bilayer substrate cases. Moreover, to see the influence of reducing symmetry of the two short bars we break the symmetry of the metasurface by decreasing the width of a short bar to *w*_1_ = *p*/6 (green) and compare it with *w*_1_ = *p*/5 at *d* = 110 nm (sky-blue) as shown in Figure 3e. It is observed that the q-BIC shifts to shorter wavelengths due to the reason that the effective dielectric constant of the structure decreases as shown in Figure 3e green curve. The influence of the geometric parameters of the short bars also confirm that the BIC is accumulated by the two short bars and long bar act as a driving parameter.

Since, we have different thickness and polarization cases which can make various combinations. However, we select the structure with infinite substrate and PMMA, and extend our simulations to nonlinear regime by using the excited near-field in van der Waals metasurface. The induced nonlinear polarization in few-layers metasurface is calculated by incident applied beam of fundamental waveband as:

$P_{i}\left(2\omega_{sh}\right)=\varepsilon_{0}\underset{j}{\sum }\underset{k}{\sum }X_{\mathrm{ijk}}^{\prime \prime }E_{j-sh}\left(\omega \right)E_{k-sh}\left(\omega \right)$ , where $X_{\mathrm{ijk}}^{\prime \prime }$ is second order nonlinear susceptibility tensor, which is $X^{\prime \prime }=270\;\text{pm}/\text{V}$ , and $X^{\prime \prime }=530\;\text{pm}/\text{V}$ calculated in [46]. These values cover a range, experimentally calculated for few-layers/monolayer TMDs nanosheets. However, few theoretical calculations estimated high values [47], [48]. A bulk MoS_2_ resonator has been reported for SHG with light dependent susceptibility estimated around 220 pm/V [33]. A WS_2_ based SHG has been reported previously with different resonator thickness. Our structure sustains BIC from ∼31 to ∼33 stacked layers which are odd layers in both case, where the thickness of monolayer is 0.65 nm (sometime varies) as mentioned in Ref. [46]. For SHG we set the thickness of meta-atoms to 33 layers to ensure the crystal symmetry breaking. The SHG efficiency is calculated by using the expression: $\eta_{sh}=\frac{P_{sh}}{P_{in}}$ , where *P*_in_ is the incident power.

**Figure 4:**
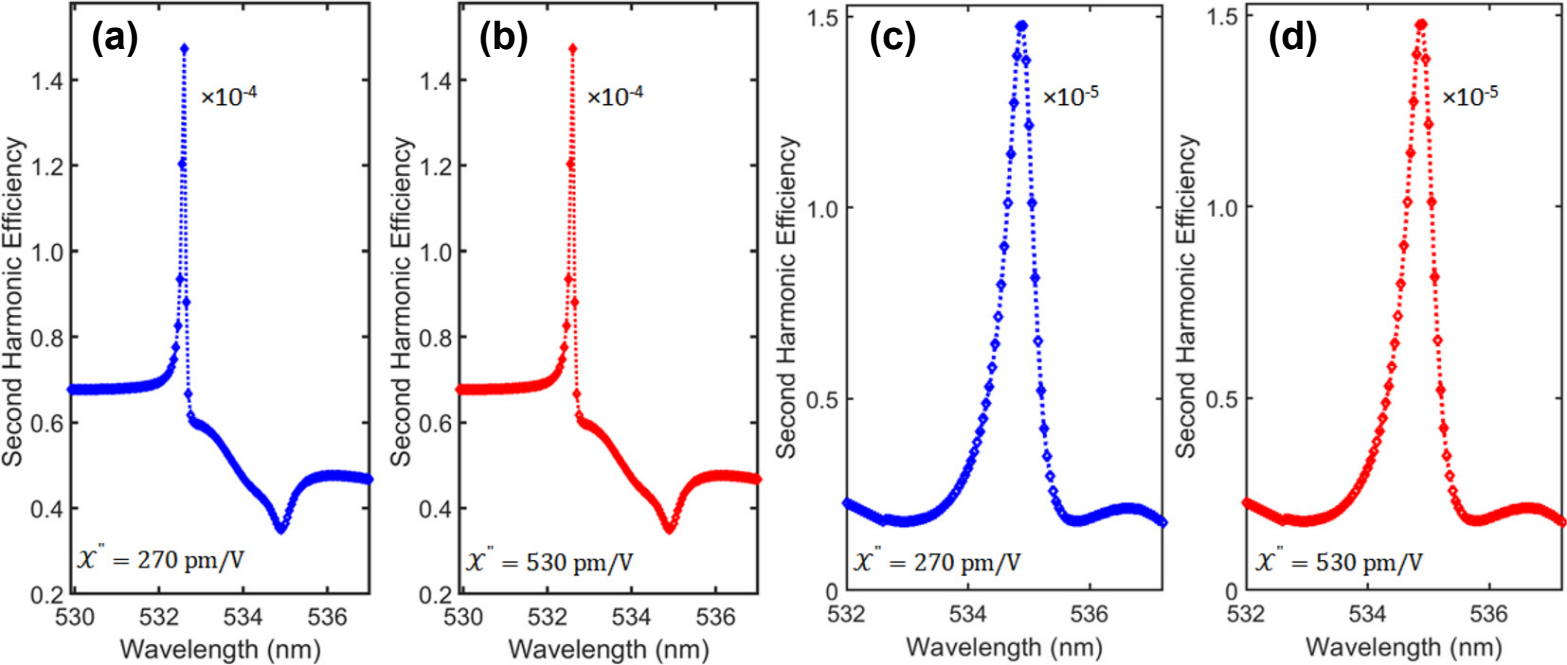
SHG conversion efficiency at displacement *d* = *p*/4.5 around BIC as a function of wavelength for (a) X′′=270pm/V (b) X′′=530pm/V with *y*-polarized applied electric field and (c) X′′=270pm/V (d) X′′=530pm/V with *x*-polarized applied electric field.

First, we consider the *y*-polarized field with two different tensor values as shown in Figure 4a and b. The structure demonstrates SHG conversion efficiency of 1.47 × 10^−4^ at 2*ω* in second harmonic (around 532 nm) which is around the BIC wavelengths in fundamental wavelengths (around 1,072 nm). Next, we apply the *x*-polarized light to the same structure and calculate the SHG conversion efficiency of 1.46 × 10^−5^ around 535 nm as shown in Figure 4c and d. As the SHG change with incident power, but in this work 6 mW is applied in both *x*- and *y*-polarizations. In nonlinear calculations the structure results SHG at two different wavelengths deviates to shorter wavelengths at *y*- and *x*-polarization. Theoretically, the SHG conversion efficiency should be around the 2*ω* in the second harmonic wavelengths. However, the deviation is not so significant, and this kind of small deviations from BIC wavelengths has been observed in literature for directional lasing [49].

The theoretical SHG conversion efficiency 1.47 × 10^−4^ in our work from an ultra-thin structure is comparable to experimentally calculated 10^−5^ in GaAs when the pump power is 11.4 mW [50], and 10^−6^ to 10^−7^ in bulk MoS_2_ [33] for 6 mW incident power. For 0.88 mW incident power the SHG changed to 1.93 × 10^−4^ in *y*-polarization, which is higher than 10^−6^ in LiNbO_3_ structure experimentally calculated under 0.88 mW [51]. Since the experimental realization is not as ideal as theoretical, it is expected that, the experimental results of few-layers structure will approach the values reported in bulk counterparts. The ∼20 nm resonator thickness is comparatively too thin than previously reported work for BIC [10], [32]. The structure sustains the shape and Q-factor in both suspended and substrates cases compare to the previously reported ultra-thin in conventional dielectric structures with thickness of 53 nm made of Si. In 53 nm Si structure the shape of magnetic field profile at BIC deteriorates when placed on substrate [21]. It is worth noting that, the TMDs meta-atoms are highly tunable, it can be observed from sustaining BIC at (31–33 layers) varied resonator thicknesses. This can be highly helpful in fabrication, maintaining the BIC with variation in number of layers and achieving the experimental results with high accuracy. The advanced fabrication techniques can be used to experimentally realize our theoretical investigations as reported in [10], [34], [41]. To achieve BIC in conventional dielectric metasurfaces homogeneous background mediums have been used [11], [17], [49]. However, our van der Waals metasurface sustains the BIC in both homogeneous and non-homogeneous mediums. In addition, ultra-thin geometries will improve the efficiency of Pancharatnam-Berry metasurfaces where the effective dielectric constant limits the performance of the meta-devices after shrinking the common dielectric structure from a certain thickness. Such ultra-thin structures with high tunability, Q-factor and remarkable modes-upholding will open-up a new window for post-silicon metasurfaces.

## 4 Conclusions

In this article, we demonstrate the BIC supported by excitons and optical modes in a ∼20 nm thick van der Waals metasurface. The metasurface maintain the BIC in both homogeneous and non-homogeneous mediums with high Q-factor. The hetero-symmetric structure is highly tunable and upholds the BIC at different thickness of meta-atoms. BIC and q-BIC can be achieved at different polarizations including left and right circularly polarized, *x*- and *y*-polarized applied beam. SHG conversion efficiency of 1.47 × 10^−4^ is calculated around BIC wavelength which is higher than reported in literature. The metasurface maintain the magnitude of SHG efficiency at *x*-polarized applied beam. The van der Waals metasurface will help to further miniaturize the meta-atoms for both linear and nonlinear nanophotonic applications [52], [53] and will provide a plat-form for next-generation metasurfaces.
